# Use of 3-D Models for Surgical Planning of a Malunion in a Dog

**DOI:** 10.1155/2022/3813525

**Published:** 2022-03-25

**Authors:** Norihiro Muroi, Kiyohisa Fujii, Masakazu Shimada, Nobuo Kanno, Yasuji Harada, Yasushi Hara

**Affiliations:** Department of Veterinary Surgery, Nippon Veterinary and Life Science University, Musashino-shi, Tokyo, Japan

## Abstract

**Background:**

An 8-year-old, 18.9 kg, male, intact Kai Ken with a femoral shaft fracture experienced recurrent implant breakage after two fracture reductions using an internal fixator.

**Objectives:**

This case report is aimed at using a three-dimensional (3-D) printer to diagnose residual femoral rotational deviation. Implant failures and malunion occurred after two attempts at synthesis. Thus, a 3-D model was designed for preoperative planning of a third surgery.

**Methods:**

To evaluate the alignment in the postoperative state after the second surgery, we removed a broken plate from the affected limb. Subsequently, a computed tomography image produced a bone replica using 3-D printing. The distal fragment was fixed and rotated externally by 42°. In addition to correcting the rotational deformity of the femur, we used an intramedullary pin and two locking plates to stabilize the proximal and distal femoral fracture segments.

**Results:**

The bone union was confirmed four months after surgery, and no postoperative complications were observed 11 months after surgery.

**Conclusion:**

3-D printing is a valuable tool that increases the accuracy of presurgical planning.

## 1. Introduction

In small animals, understanding the shape of fracture fragments using orthogonal radiographs and computed tomography (CT) images is essential for the surgical planning and postoperative evaluation of orthopedic surgery. However, accurate assessments of the characteristics of fracture fragments in three dimensions can be challenging to perform with two-dimensional images on a flat screen. Therefore, three-dimensional (3-D) rendering from CT data has been recently reported to be a valuable method for preoperative planning in human medicine and small animal veterinary medicine [1–4]. The life-sized 3-D-printed bone replica allows better visualization of fractures in multiple directions, improving the quality of surgical planning, e.g., by enabling accurate contouring of implants and shortening the operation time. The use of 3-D printing for preoperative planning was recently described in a dog with complicated comminuted femoral fracture repair [5]. While prior studies have reported the use of 3-D printing for preoperative planning, in the present report, we describe 3-D printing to replicate improper femoral alignment after two failed surgical reductions in a dog with a femoral diaphyseal comminuted fracture. This case report is aimed at describing that the evaluation of bone morphology using a 3-D bone model created by a printer helped confirm the nature of the deformity of the femur caused by internal fixation due to fracture reduction.

## 2. Case Presentation

An 8-year-old, 18.9 kg, male intact, Kai Ken had previously been diagnosed with a left femoral diaphyseal comminuted fracture and had undergone surgical fracture repair with an internal fixator at the first private animal clinic. However, the dog experienced implant breakage 12 days after surgery, and the second surgical repair was performed. Three months after the second surgery, the dog exhibited nonweight-bearing right hindlimb lameness. The owner brought the dog to a different private animal clinic, and implant breakage was confirmed by radiography. The dog was referred to our university for evaluation of the broken plate. Unfortunately, information regarding the initial fracture configuration and the surgical procedure details at the first private animal clinic was unavailable.

### 2.1. Treatment Plan

**Figure 1:**
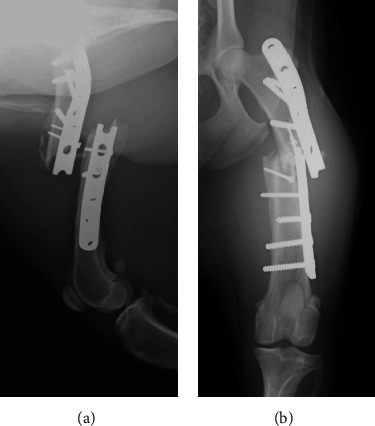
Preoperative radiographs. (a) Lateral view. b) Anterior-posterior view.

At the initial presentation at the Veterinary Medical Teaching Hospital, blood tests (i.e., complete blood count, thyroxine, thyroid-stimulation hormone, cortisol, C-reactive protein, and serum chemistry panel) were performed to rule out concurrent conditions that would cause delayed bone union. Based on the test results, diabetes mellitus, hypothyroidism, Cushing's disease, metabolic disease, liver disease, and kidney disease were ruled out. Moreover, thoracic and abdominal radiographs and abdominal ultrasonography showed unremarkable findings. However, radiographs of the left hindlimb revealed that the plate used for the femoral shaft fracture was broken at a screw hole around the fracture line, and the distal femur was dislocated (Figure 1). Furthermore, no evidence of bone tumor characterized by excessive bone resorption or formation was found on radiographs. In addition to the screws used for the plate holes, there were two individual screws on the femoral shaft, suggesting that the dog initially had a reducible comminuted fracture of the femoral diaphysis, which was treated using the lag screw technique.

Considering the dog's age and his history of repeated plate breakage, improper reduction, bone tumor, and abnormal bone density were included in the differential diagnosis. Thus, the broken plate was surgically removed before surgical fracture repair, and a CT scan was performed to evaluate bone tumor and bone density. In addition, a life-sized bone replica was created using 3-D printing from a CT image to replicate the second surgical fracture repair.

### 2.2. Surgical Removal of the Broken Implant

Nine days after the second plate breakage, the femur was laterally approached, and all implants were surgically removed. After removing the implants, a CT scan (Aquilion PRIME TSX-303A, Toshiba Medical Systems Corporation, Japan) was performed to evaluate both femurs.

### 2.3. Anesthesia

Atropine sulfate (0.04 mg/kg intravenously (IV)) and midazolam hydrochloride (0.2 mg/kg IV) were administered as a preanesthetic medication. Propofol (4 mg/kg IV) was infused for anesthesia induction, and the dog was intubated. Isoflurane was used to maintain the surgical plane of anesthesia. Analgesia was also administered with epidural anesthesia (bupivacaine hydrochloride: 1 mg/kg, EI) and fentanyl citrate (3-15 *μ*g/kg/h intravenous continuous rate infusion (CRI)). Cefazolin sodium (25 mg/kg IV) was administered 30 min before surgery and every 90 min intraoperatively until 2 h after surgery [6].

### 2.4. Evaluation of the Broken Plate and Other Screws and Bone Fracture after the Procedure

The broken plate was a 3.5 mm dynamic compression plate (148 × 10 mm; thickness, 3.0 mm; 12 screw holes; 12 mm interval between each hole; 3.5 mm narrow plate II; Mizuho Corporation, Japan). Eight plate screws (3.5 mm cortical screw; Mizuho Corporation, Japan) and two lag screws (2.7 mm cortical screw; Mizuho Corporation, Japan) were surgically removed with the broken plate. The proximal fragment was stable after removing the lag screws, suggesting that bone union had been completed.

### 2.5. Computed Tomography Scan

The CT scan acquisition settings were as follows: X-ray tube voltage, 120 kV; X-ray tube current, 300 mA; scan speed, 0.5 s; and slice thickness, 0.5 mm. Based on the CT scan data, femoral torsion angle (FTA, reference value [7]: mean ± SD, 19.6° ± 7.9°), femoral inclination angle (FIA, reference value [8]: mean ± SD, 134.0° ± 5.2°), and anatomical lateral distal femoral angle (aLDFA, reference value [7]: mean ± SD, 98.8° ± 3.3°) of the usual right limb were 22°, 130°, and 93°, respectively (Figure 3). Phantom-based (B-MAS200; Kyotokagaku, Kyoto, Japan) qualitative CT (QCT) acquisition was performed to measure the bone mineral density (BMD) of both femurs [9, 10]. The BMD of the femoral cortical bone of the affected limb (left) was 1098 mg/cm^3^, whereas that of the right femur was 1001.5 mg/cm [3].

### 2.6. Three-Dimensional Printing

**Figure 2:**
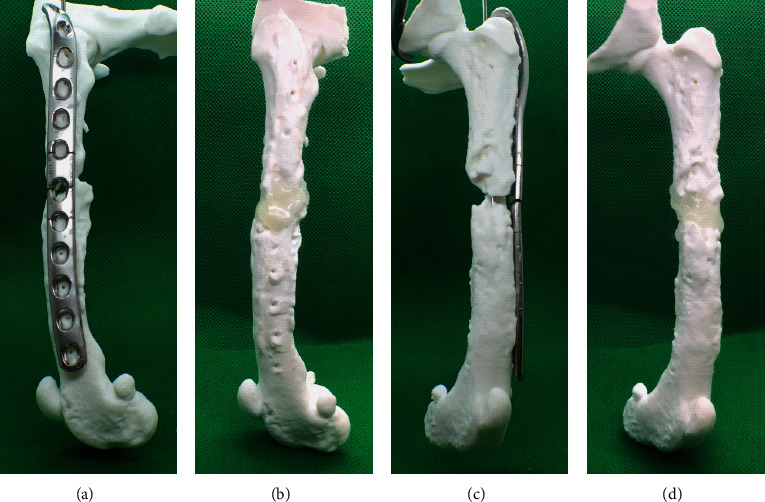
A model created with a three-dimensional printer. The alignment of the femur was confirmed by reproducing the femur with the plate and screws fixed at the time of the previous surgery. Although difficult to divide in the lateral view, the distal fracture fragments can be observed to be fixed outward in the anterior-posterior view. (a) Lateral view with the plate fixed. (b) Lateral view without the plate. (c) Anterior-posterior view with the plate fixed. (d) Anterior-posterior view without the plate.

A life-sized 3-D bone replica was reproduced according to previous reports [11–13]. The CT images were converted to multiplanar reconstruction (MPR) images using software (AZE VirtualPlace, AZE Corporation, Japan). Subsequently, based on the MPR images, the left femur was printed using a fused deposition modeling 3-D printer (L-DEVO M3145TP, Fusion Technology, Japan). The material used was PolyMax PLA (Polymaker, Shanghai). Finally, the plate and screws removed from the patient were applied to the 3-D bone replica to reproduce the reduced condition after the second surgery (Figure 2).

### 2.7. Evaluation of the 3-D Bone Replica

**Figure 3:**
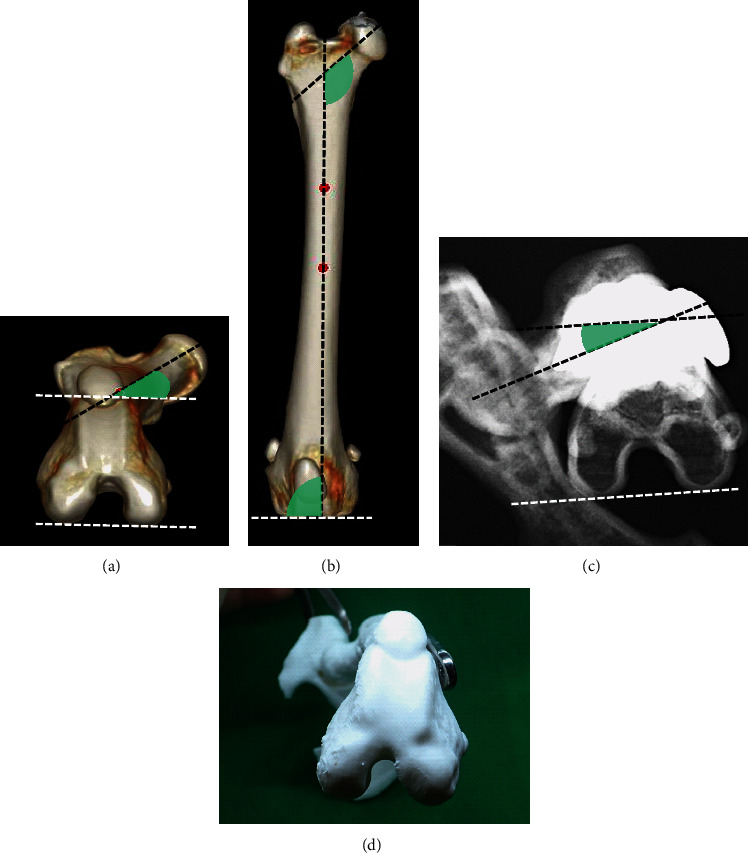
FTA, FIA, and aLDFA measurements in the unaffected and affected limbs with fractures. The unaffected limb was evaluated using CT images. In addition, the affected limb was assessed using radiographs of the model output using a 3-D printer. The affected limb was fixed with 42° external rotation compared to the unaffected limb. (a) FTA measurements in the CT image of the unaffected limb. (b) FIA and aLDFA measurements in the CT image of the unaffected limb. (c) FTA measurements in radiographic images of a bone model that replicates a predicted previous surgery. (d) Photographs of bone models taken in the same direction as the radiograph. FTA: femoral torsion angle; FIA: femoral inclination angle; aLDFA: anatomical lateral distal femoral angle; CT: computed tomography.

The second surgical reduction was reproduced using the bone replica. The FTA, FIA, and aLDFA of the affected femur (left) were shown to be -20°, 130°, and 101°, respectively. In addition, the distal left femoral fragment was rotated 42° externally compared to the right femur. These findings suggested that the distal segment was fixed in a state of external rotation against the proximal segment (Figure 3).

### 2.8. Surgical Planning

The morphometric results of the affected limb's femur indicated that only the FTA was abnormal. Furthermore, since the surgery is aimed at correcting the rotational deformity of the femur, we simulated the procedure using 3-D bone replicas. This allowed us to confirm that using intramedullary pins to fix the fracture fragments during the first fracture reduction step reduces fragment displacement in the anterior-posterior and mediolateral directions, thus allowing for easier fixation in the rotational direction. Therefore, the plate-rod method was used in this surgery. In addition, the shape of the femur was complex, and it was difficult to accurately correct its morphology based on the position of the femoral head, greater trochanter, and patella. Consequently, we used the screw holes of the second surgery as landmarks to correct the direction of femur rotation. Specifically, an intramedullary pin was placed on the 3-D bone replica, and the femur was subsequently rotated to match the contralateral FTA. The rotational distance was subsequently measured using a bone chisel to mark the cortical bone of the femur and a caliper to measure the distance of movement.

### 2.9. Fracture Repair

Three days after surgical removal of the implants, fracture repair was performed. A lateral approach to the left femur was made, and the fracture fragment was exposed. Debridement was performed on the edges of the proximal and distal segments. The proximal and distal femoral segments were reduced by a retrograde technique using a 2.4 mm Steinmann Pin. In addition to measuring the femoral rotation distance during preoperative planning, the morphology of the femoral rotation angle was confirmed by placing Steinmann Pins on the distal and proximal fracture fragments, respectively. In the distal fracture fragment, the pin was inserted from the center of the lateral epicondyle to the center of the medial epicondyle. Conversely, in the proximal fracture fragment, the pin was inserted from the lateral side of the greater trochanter to the distal femoral neck parallel to the cranial surface of the proximal femoral cortical bone. After correcting the direction of femur rotation, the fragments were stabilized using a plate-rod fixation technique with a 3.5 mm string of pearls plate (SOP; Orthomed Ltd., Huddersfield, West Yorkshire, United Kingdom), 3.5 mm cortex screws on the lateral side of the femur (proximal bone fragment, four screws; distal bone fragment, four screws), a 2.7 mm locking compression plate (LCP; DePuy Synthes Inc., West Chester, Pennsylvania, United States), and 2.7 mm locking head screws (LHS; DePuy Synthes Inc., West Chester, Pennsylvania, United States) on the craniolateral side of the femur (proximal bone fragment, two screws; distal bone fragment, three screws). Appropriate alignment and positioning of the implants were confirmed using postoperative radiographs. The postoperative FTA, FIA, and aLDFA of the left limb were 27.8°, 130°, and 100°, respectively. The bone fragment removed during debridement was subjected to a bacterial culture test, and coagulase-negative *Staphylococcus* was detected.

### 2.10. Postoperative Management

The duration of hospitalization after fracture repair was 14 days. During hospitalization, exercise was restricted. Cefazolin sodium (25 mg/kg) was administered twice daily intravenously for the first four days and subcutaneously (SQ) for the remaining days. Simultaneously, enrofloxacin (5 mg/kg SQ once daily) was administered. For pain management, fentanyl citrate (2 *μ*g/kg/h intravenous CRI) was continued until 8 h after surgery. In addition, a fentanyl patch (8.4 mg/head, transdermal preparation) was applied, and fentanyl citrate CRI was discontinued after 8 h. A Robert–Jones bandage was applied to the limb to reduce postoperative inflammation and edema, and the dressing was changed every day for six days during hospitalization. Dermatitis and decubitus ulcers were checked twice a day until discharge. The dog started toe-touching four days after surgery.

### 2.11. Outcome

**Figure 4:**
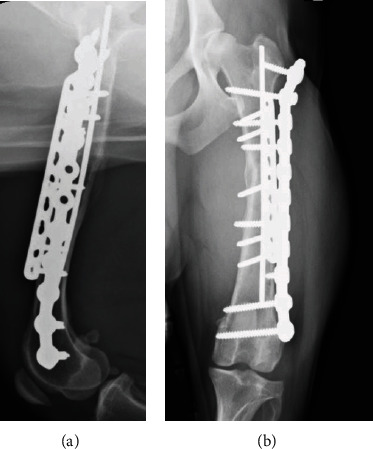
Postoperative radiographs. Postoperative bone healing was good, and cortical bone continuity was observed four months postoperatively, confirming that bone healing had been achieved. (a) Lateral view at four months after surgery. (b) Anterior-posterior view at four months after surgery.

The dog was brought to the Veterinary Medical Teaching Hospital for follow-up evaluation 30 days after surgery. The orthopedic evaluation revealed mild weight-bearing lameness. Radiographic assessment of the hindlimbs was performed monthly for four months after surgery. The ground reaction force (GRF) was simultaneously evaluated by measuring peak vertical force/body weight (PVF/BW) using a force plate (Advanced Mechanical Technology, Inc., Watertown, MA, USA). Radiographic examination revealed callus formation and bone union one and four months after surgery, respectively (Figure 4). Short-term postoperative complications, such as plate breakage or infection, were not observed.

Four months after surgery, radiographic examination and measurement of GRF were performed every 2–3 months for 11 months after surgery. Weight-bearing had started increasing three months after surgery. The PVF/BW of the normal limb (right) and the affected limb (left) were 63.7% and 58.3%, respectively, suggesting that the weight-bearing function in the affected limb had recovered 11 months after surgery. Follow-up examinations were discontinued 11 months after surgery per the owner's request.

## 3. Discussion

3-D models are used worldwide as a presurgical planning tool for many cases. In this case, we used models of both femurs to understand and plan the preoperative and transoperative strategies to be used. Two plates and an intramedullary pin (plate-rod technique) were used to correct the rotational deviation and fix corticotomy.

In the case of a reducible comminuted fracture, the anatomical reduction of the fragments involved the segments: proximal and distal fragments are crucial. However, achieving precision during surgery can be challenging. The proximal femoral segment is often rotated externally because of the gemellus muscle and internal and external obturator muscles [14]. Thus, improper alignment has been reported after surgical reduction in dogs with comminuted fractures of the femoral diaphysis [15]. This report describes the use of 3-D printing from CT images to evaluate fragment alignment after implant breakage in a dog with a femoral shaft comminuted fracture. In the present case, the life-sized bone replica assessment revealed that the distal fragment had been fixed at 42° externally before surgery. It was difficult to find any improper alignment of the femur in the rotational angle on the radiographs after plate failure. Considering the dog's body weight, the 3.5 mm plate used in the second surgery may have been suitable [16]. However, because the dog experienced plate breakage twice, a stronger fixation was chosen at our hospital. The proximal and distal fragments were stabilized with an intramedullary pin, and two locking plates were laterally applied. A double-plate fixation method has been reported to provide increased stability against rotation and flexion [17–20]. Consequently, bone union was confirmed four months after surgery, and no postoperative complications were observed 11 months after surgery.

The fracture configuration in the femur can be evaluated by measuring FTA, FIA, and aLDFA on radiographs or CT images. In the present case, FTA (-20°) of the affected femur was 42° less than that of the healthy femur (reference value: mean ± SD, 19.6° ± 7.9°). The FIA (130°) and aLDFA values of both femurs (101°) were comparable to the reference values. Implant breakage can result from fatigue failure of an implant due to repeated bending stress on an implant [21]. At the time of weight-bearing on the hindlimbs, compression occurs on the medial femoral cortex, whereas tension occurs in the lateral femoral cortex [16]; hence, the femur tends to bend toward the medial side. Thus, a buttress plate for comminuted fracture requires strength to control the bending toward the medial side. In the present case, the screws and the plate were of appropriate size for the dog's weight. Therefore, we suspected that the treatment failure was due to fatigue failure of the plate (in addition to the bending stress on the plate, torsional stress due to rotational deformation of the femur may be present) or bacterial infection. If plate breakage was caused by fatigue failure due to a rotational deformation of the femur, precise reduction of the proximal and distal fragments would have been more crucial than stabilization with a double plate. In addition, the fracture fragments after implant breakage were dislocated. Therefore, a radiographic examination cannot accurately evaluate fracture configuration. In the present case, 3-D CT imaging and a life-sized bone replica allowed us to diagnose the improper alignment that radiographs did not show. Bone replicas created by 3-D printers can be evaluated in three dimensions, making them more useful than radiographic images in assessing bone morphology before refracture. Thus, 3-D printers are valuable tools to assess bone morphology before refracture.

Previously, the goal of applying a 3-D printer in veterinary orthopedics was to design a preoperative plan in order to reconstruct the comminuted fracture in the joint [5]. In the present case, we employed a 3-D printer to confirm whether the fracture was reduced correctly in the second surgery. Generally, when the ORIF method was used for the reduction of femoral fractures, palpable anatomical landmarks, including the greater and lesser trochanters, and lateral and medial supracondylar tuberosities of the femur, were used to understand the positional relationship of the fragments. However, in some cases with a chronic process after the initial fracture, the abundant callus and fibrous tissue formed around the fracture ends might disturb the confirmation of the bony landmarks at the operation. Moreover, the muscle may show contracture because of previous multiple operations, rendering it difficult to reconstruct the correct alignment of the femur. Under these circumstances, given that the surgeon missed the actual cause of failure of the previous operation, he had judged the cause of implant breakage as the inadequate selection of implant size, which was too small or insufficiently stiff for the fracture. The surgeon might have fixed the fracture using a plate of a larger size at the same location, as in the previous operation. The actual cause of implant breakage, in this case, could not be clarified, and it was considered that inadequate reduction between the proximal and distal fragments with rotational misalignment at the initial surgery might be a possible cause. The results obtained in this case suggest that prior 3-D analysis and the use of the correct reduction technique reduced bias in the planning and resolution of the described case.

## 4. Conclusion

A life-sized bone replica produced by 3-D printing has been reported to allow surgeons to assess fracture configuration preoperatively and facilitate surgical planning, including countering a plate before surgery, leading to shorter operation times [22]. In the present case report, the preoperative 3-D printing of the affected bone (left femur) allowed for orthopedic surgical planning, without bias, and, combined with the correct technique (plate-rod), we achieved with success the ultimate goal of treatment.

## Data Availability

The data that support the findings of this study are available from the corresponding author upon reasonable request.
